# p53 Regulates Mitochondrial Dynamics in Vascular Smooth Muscle Cell Calcification

**DOI:** 10.3390/ijms24021643

**Published:** 2023-01-13

**Authors:** Kanchan Phadwal, Qi-Yu Tang, Ineke Luijten, Jin-Feng Zhao, Brendan Corcoran, Robert K. Semple, Ian G. Ganley, Vicky E. MacRae

**Affiliations:** 1 The Roslin Institute and Royal (Dick) School of Veterinary Studies, University of Edinburgh, Midlothian EH25 9RG, UK; 2 Centre for Cardiovascular Science, Queens Medical Research Institute, University of Edinburgh, 47 Little France Crescent, Edinburgh EH16 4TJ, UK; 3 MRC Protein Phosphorylation & Ubiquitylation Unit, Sir James Black Centre, University of Dundee, Dundee DD1 5EH, UK

**Keywords:** vascular smooth muscle cells, arterial calcification, mitochondrial dynamics, senescence

## Abstract

Arterial calcification is an important characteristic of cardiovascular disease. It has key parallels with skeletal mineralization; however, the underlying cellular mechanisms responsible are not fully understood. Mitochondrial dynamics regulate both bone and vascular function. In this study, we therefore examined mitochondrial function in vascular smooth muscle cell (VSMC) calcification. Phosphate (Pi)-induced VSMC calcification was associated with elongated mitochondria (1.6-fold increase, *p* < 0.001), increased mitochondrial reactive oxygen species (ROS) production (1.83-fold increase, *p* < 0.001) and reduced mitophagy (9.6-fold decrease, *p* < 0.01). An increase in protein expression of optic atrophy protein 1 (OPA1; 2.1-fold increase, *p* < 0.05) and a converse decrease in expression of dynamin-related protein 1 (DRP1; 1.5-fold decrease, *p* < 0.05), two crucial proteins required for the mitochondrial fusion and fission process, respectively, were noted. Furthermore, the phosphorylation of DRP1 Ser637 was increased in the cytoplasm of calcified VSMCs (5.50-fold increase), suppressing mitochondrial translocation of DRP1. Additionally, calcified VSMCs showed enhanced expression of p53 (2.5-fold increase, *p* < 0.05) and β-galactosidase activity (1.8-fold increase, *p* < 0.001), the cellular senescence markers. siRNA-mediated p53 knockdown reduced calcium deposition (8.1-fold decrease, *p* < 0.01), mitochondrial length (3.0-fold decrease, *p* < 0.001) and β-galactosidase activity (2.6-fold decrease, *p* < 0.001), with concomitant mitophagy induction (3.1-fold increase, *p* < 0.05). Reduced OPA1 (4.1-fold decrease, *p* < 0.05) and increased DRP1 protein expression (2.6-fold increase, *p* < 0.05) with decreased phosphorylation of DRP1 Ser637 (3.20-fold decrease, *p* < 0.001) was also observed upon p53 knockdown in calcifying VSMCs. In summary, we demonstrate that VSMC calcification promotes notable mitochondrial elongation and cellular senescence via DRP1 phosphorylation. Furthermore, our work indicates that p53-induced mitochondrial fusion underpins cellular senescence by reducing mitochondrial function.

## 1. Introduction

Arterial calcification involves the deposition of hydroxyapatite crystals within blood vessels including the aorta, carotid and femoral arteries [1]. The process of arterial calcification shares key similarities with physiological bone formation [2,3,4]. Indeed, numerous reports have demonstrated that in a calcified environment, vascular smooth muscle cells (VSMCs), the principal cell type underpinning arterial calcification, can transdifferentiate to osteoblast-like cells [5,6]. Furthermore, inorganic phosphate (Pi) accelerates this transition, as evidenced by the upregulation of osteogenic markers, such as osteocalcin (OCN), sodium-dependent phosphate co-transporter (PiT-1) and tissue-nonspecific alkaline phosphatase (TNAP) [7]. Arterial calcification additionally proceeds via the reciprocal loss of recognised calcification suppressors, including fetuin, inorganic pyrophosphate (PPi) and matrix Gla protein (MGP) [8]. Furthermore, there is the growing evidence that elevated Pi levels can also induce VSMC senescence, with human aortic VSMCs showing upregulated expression of p53 and its downstream target p21, in combination with elevated senescence-associated β-galactosidase (β-gal) activity [9]. Indeed, senescent VSMCs appear to play an important role in mediating the process of arterial calcification [10,11,12].

Mitochondria are crucial bioenergetics hubs within cells, generating toxic reactive oxygen species (ROS) following exposure to oxidative stress [13]. Mitochondrial physiology and function are likely to be affected by this pathological process. Impaired mitochondria function, oxidative stress, modulated mitochondrial metabolism and mitochondrial DNA damage have been noted in disorders that display arterial calcification including type 2 diabetes, end stage renal disease and atherosclerosis [14]. Furthermore, cells undergoing senescence have been reported to show altered mitochondrial function [15,16]. Recent studies by our laboratory have shown that calcifying VSMCs show reduced oxygen consumption rate, ATP-linked respiration and maximal respiration, suggestive of dysregulated mitochondrial activity [17]. However, molecular mechanisms linking mitochondrial dysfunction, cellular senescence and arterial calcification have yet to be determined. In this study, we tested the hypothesis that the senescence regulator p53 plays a functional role in arterial calcification through the regulation of mitochondrial dynamics.

## 2. Results

### 2.1. High Phosphate Treatment Alters Mitochondrial Dynamics in VSMCs

**Figure 1:**
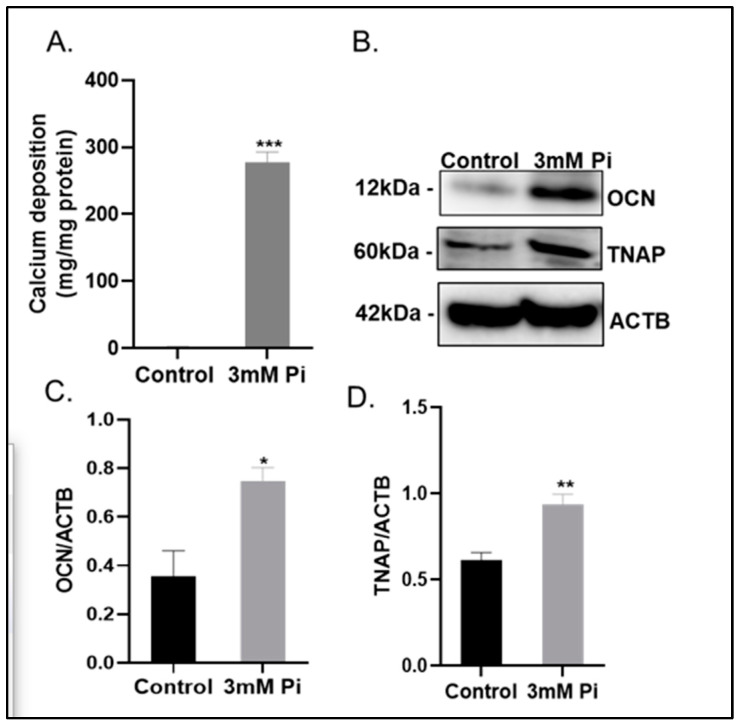
Calcification of VSMCs in vitro. (**A**) Calcium content (mg/mg protein) of VSMCs (n = 6). (**B**) Representative image of immunoblotting and associated density quantification of (**C**) Osteocalcin (OCN) (n = 3) and (D) tissue-nonspecific alkaline phosphatase (TNAP) (n = 3) protein expression compared with ACTB (β-actin). Data shown as mean +/− S.E.M * *p* < 0.05; ** *p* < 0.01; *** *p* < 0.001 compared to control.

Treatment with 3 mM Pi produced an increase in VSMC calcium deposition by day 14 compared with cells cultured in control medium (*p* < 0.001; Figure 1A). In alignment with recent reports from our laboratory, 3 mM Pi-induced osteogenic transdifferentiation in VSMCs [18,19], as demonstrated by the upregulated protein expression of OCN (2.1-fold increase, *p* < 0.01; Figure 1B,C) and TNAP (1.5-fold increase, *p* < 0.05; Figure 1B,D).

**Figure 2:**
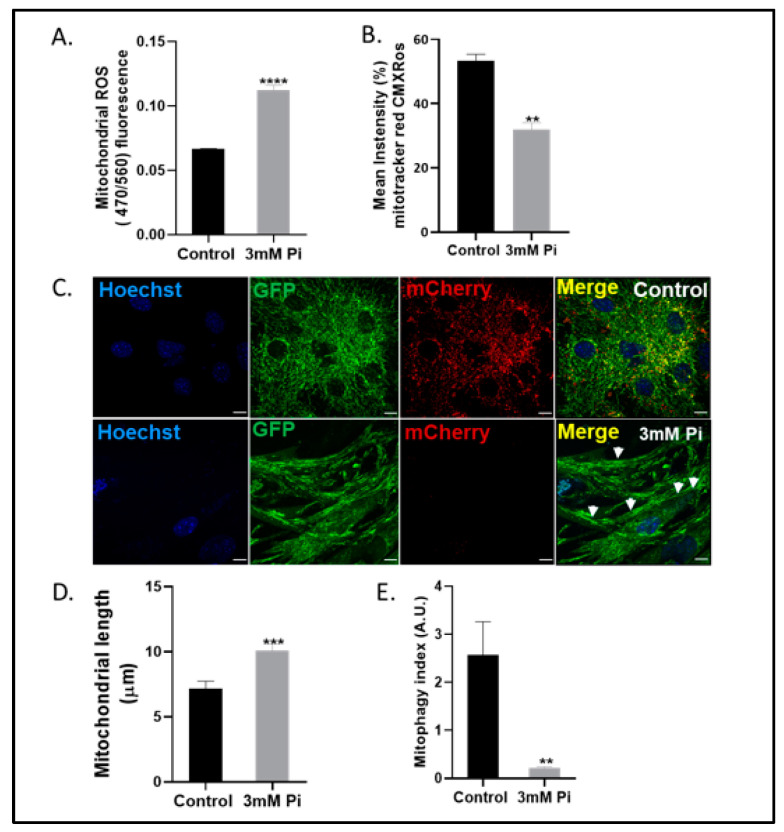
Mitochondrial elongation and dysfunction in calcified VSMCs. Quantification of (**A**) mitochondrial reactive oxygen species (ROS) (470/560 nm) and (**B**) mitochondrial membrane potential by mitotracker red CMXRos (mean intensity). (**C**) Representative confocal images of mito-QC VSMCs (white arrows show hyperfused mitochondria). Quantification of (**D**) mitochondrial length (µM) and (**E**) Mitophagy index. Data shown as mean +/− S.E.M. (n = 6) ** *p* < 0.01; *** *p* < 0.001; **** *p* < 0.0001 compared to control. Scale bars = 10 μm.

Our previous work has shown that VSMCs cultured in calcification medium have impaired respiratory status, marked with low ATP levels and reduced oxygen consumption rate [17]. These data suggest altered mitochondrial function in the arterial calcification process. We now extend these findings and report an increase in mitochondrial reactive oxygen species (ROS) production (1.83-fold increase, *p* < 0.001; Figure 2A), with a concomitant decrease in mitochondrial membrane potential (as determined by MitoTracker Red CMXRos, 1.6-fold increase, *p* < 0.01; Figure 2B) in calcified versus control VSMCs.

We next sought to visualize mitochondrial morphology and assess mitophagy (a selective type of autophagy that necessitates the removal of dysfunctional mitochondria) [20,21] in calcified VSMCs isolated from mito-QC transgenic mice. Mito-QC mice utilize a binary fluorescence system whereby a globally expressed tandem mCherry–GFP tag targets mitochondria [22], resulting in these organelles fluorescing red and green within mito-QC mice. Following capture by lysosomes, the low pH reduces the fluorescence of GFP, resulting in mitochondria showing as mCherry foci. Quantification of confocal microscopy images confirmed that calcified mito-QC VSMCs contained hyperfused mitochondria of increased length (1.6-fold increase, *p* < 0.001; Figure 2C,D), with an associated reduction in the mitophagy index (9.6-fold decrease, *p* < 0.01; Figure 2E) measured as mCherry-only puncta compared to control cells. Hyperfused mitochondria in calcified VSMCs were also observed using translocase of mitochondrial outer membrane protein (TOM20) staining (Figure S1 (Supplementary Materials)). Together, our data show for the first time that mitochondria within calcified VSMCs are elongated, damaged and do not undergo the process of mitophagic degradation.

### 2.2. Regulators of Mitochondrial Fusion and Fission Drive Mitochondrial Elongation in Calcified VSMCs

**Figure 3:**
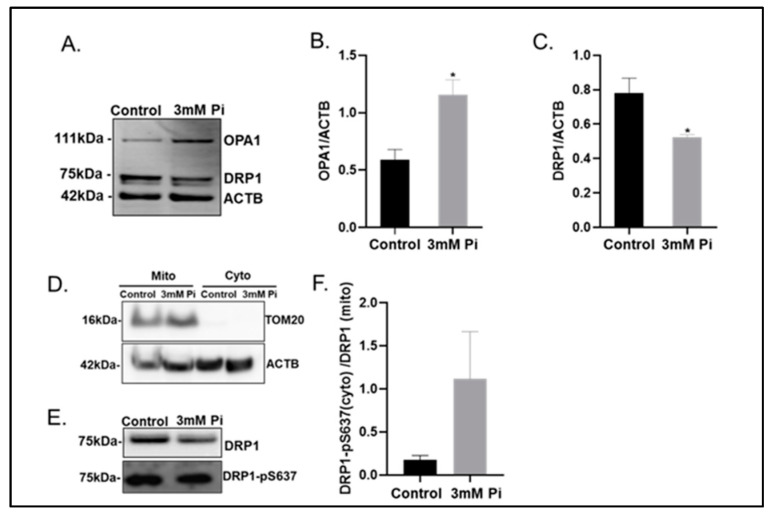
Calcified VSMCs show modulated Optic Atrophy Protein 1 (OPA1) and Dynamin-related protein 1 (DRP1) protein expression (**A**) Representative immunoblotting of whole cell lysates and associated quantification of (**B**) OPA1 and (**C**) DRP1 expression compared with ACTB (β-actin). (**D**) Representative immunoblotting of mitochondrial and cytoplasmic fraction with TOM20 and ACTB (β-actin). (**E**) Representative immunoblotting of mitochondrial expression of DRP1 and cytoplasmic expression of DRP1– pS637 associated quantification of (**F**) ratio of cytoplasmic DRP1- pS637/mitochondrial DRP1. Data shown as mean +/− S.E.M (n = 3) * *p* < 0.05 compared to control.

The capacity of mitochondria to alter their morphology is linked to the intricate processes of fission or fusion. We therefore next sought to elucidate the protein expression levels of optic atrophy protein 1 (OPA1) and dynamin-related protein 1 (DRP1), two crucial proteins needed for mitochondrial fusion and fission, respectively [23,24]. Immunoblotting studies revealed an enhanced expression of OPA1 (2.1-fold increase, *p* < 0.05; Figure 3A,B) and a reduced expression of DRP1 (1.5-fold decrease, *p* < 0.05; Figure 3A,C) in whole cell lysates produced from VSMCs cultured in calcification versus control medium.

Next, we undertook mitochondrial fractionation (enrichment confirmed by TOM20 expression, Figure 3D), to determine DRP1 expression levels specifically associated with mitochondria. Reduced expression of DRP1 was also observed in the enriched mitochondrial fraction produced from calcified versus control VSMCs (Figure 3E). However, DRP1 function is also modulated by a number of post-translational modifications, such as phosphorylation. In particular, the phosphorylation of DRP1 at Ser637 has been shown to impede its mitochondrial translocation, thus promoting mitochondrial elongation [15]. Consistent with these reports, the ratio of cytoplasmic DRP1 Ser637 phosphorylation to mitochondrial DRP1 was notably elevated in calcified VSMCs (Figure 3E,F).

Together these data strongly suggest that mitochondrial elongation in calcified VSMCs is driven by (i) increased expression of the fusion protein OPA1, (ii) loss of the fission protein DRP1 required for mitophagic degradation and (iii) enhanced mitochondrial fusion via inhibitory phosphorylation of DRP1 at Ser637.

### 2.3. Calcified VSMCs Show Increased Expression of Senescence Markers

**Figure 4:**
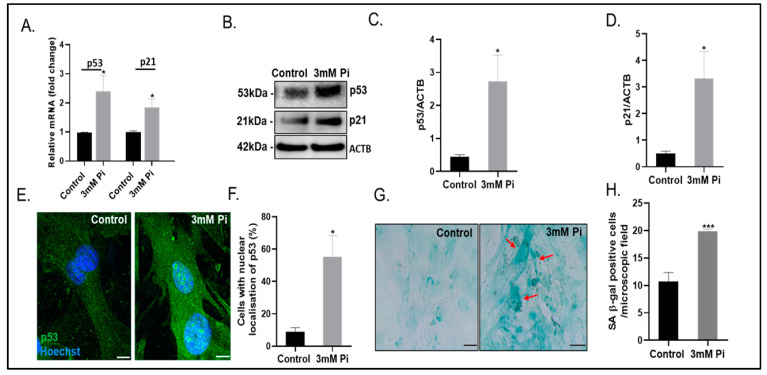
Increased senescence in calcified VSMCs (**A**) Fold change in mRNA expression of p53 and p21. (**B**) Representative immunoblots and associated quantification of (**C**) p53 and (**D**) p21 compared with ACTB (β-actin). (**E**) Representative images and (**F**) associated quantification of cells with p53 staining in the nucleus (**G**) Representative images and (**H**) associated quantification of SA β-gal staining (red arrows indicate positive staining). Data shown as mean +/− S.E.M (n = 3–6) * *p* < 0.05; *** *p* < 0.001 compared to control. Scale bars = 5 μm.

Hyperfused dysfunctional mitochondria are a hallmark of senescence and are frequently observed in natural aging and diseases associated with aging [25,26,27]. We therefore next investigated if calcified VSMCs also show an upregulated expression of established senescence markers. Indeed, VSMCs cultured in calcification medium showed increased mRNA and protein expression levels of p53 (2.5-fold increase, *p* < 0.05; Figure 4A–C) and p21 (1.9-fold increase, *p* < 0.05; Figure 4A,B,D), key transcription factors involved in cell cycle regulation, DNA damage and stress [28]. More calcified cells showed the localisation of p53 in the nuclei compared to control VSMCs (5.5-fold increase, *p* < 0.05; Figure 4E,F). Furthermore, cellular senescence-associated β-galactosidase activity, a typical feature of cellular senescence, was also noted in calcified VSMCs compared to control cells (1.8-fold increase, *p* < 0.001; Figure 4G,H).

### 2.4. p53 Suppression Blunts VSMC Calcification and Senescence

**Figure 5:**
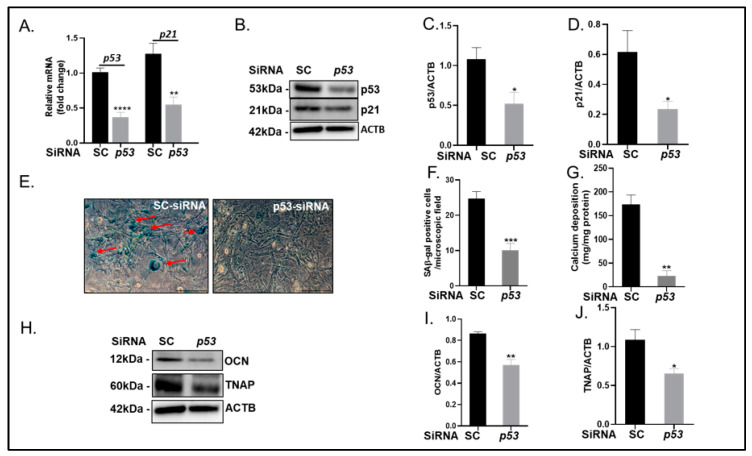
Knockdown of p53 decreases VSMC calcification and senescence. VSMCs were transfected with either scrambled (SC) or p53 siRNA and cultured with high phosphate (3mM Pi) for up to 14 days. (**A**) Fold change in mRNA expression of p53 and p21. (**B**) Representative immunoblots and associated quantification of (**C**) p53 and (**D**) p21compared with ACTB (β-actin). (**E**) Representative images and (**F**) associated quantification of SA β-gal staining (red arrows indicate positive staining). (**G**) Calcium content (mg/mg protein) of cells. (**H**) Representative immunoblots and associated quantification of (**I**) Osteocalcin (OCN) and (**J**) tissue-nonspecific alkaline phosphatase (TNAP) compared with ACTB (β-actin). Data shown as mean +/− SEM (n = 3–6) * *p* < 0.05; ** *p* < 0.01; *** *p* < 0.001; **** *p* < 0.0001 compared to control. Scale bars = 5 μm.

Increasing numbers of studies have demonstrated critical roles for the tumour suppressor p53 in regulating not only cellular senescence but also mitochondrial function [15]. To determine the functional actions of p53 within the process of VSMC calcification, we employed RNA interference to suppress p53 expression in VSMCs (2.5-fold decrease, *p* < 0.001; Figure 5A; *p* < 0.05; Figure 5B,C). In addition, p53 knockdown significantly reduced p21 mRNA (2.4-fold decrease, *p* < 0.01; Figure 5A) and protein expression (3.1-fold decrease, *p* < 0.05; Figure 5B,D). Furthermore, p53 knockdown reduced cellular senescence-associated β-galactosidase activity (2.6-fold decrease, *p* < 0.001; Figure 5E,F) and calcium deposition compared to scrambled siRNA-treated cells cultured in calcification medium (8.1-fold decrease, *p* < 0.01; Figure 5G), with an associated reduction in the protein expression of the key osteogenic molecules OCN (1.4-fold decrease, *p* < 0.001; Figure 5H,I) and TNAP (1.6-fold decrease, *p* < 0.05; Figure 5H,J).

### 2.5. Knockdown of p53 Reverses Mitochondrial Dysfunction

**Figure 6:**
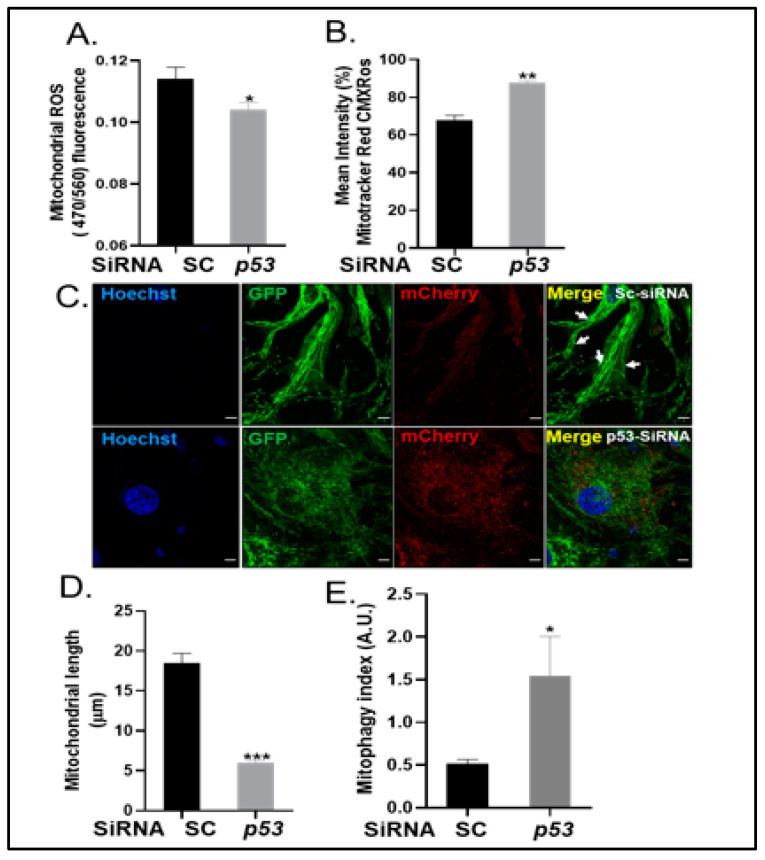
Knockdown of p53 in calcified VSMCs reduces mitochondrial length and improves mitochondrial function. VSMCs were transfected with either scrambled (SC) or p53 siRNA and cultured with high phosphate (3mM Pi) for up to 14 days. Quantification of (**A**) mitochondrial reactive oxygen species (ROS) (470/560 nm) and (**B**) mitochondrial membrane potential by mitotracker red CMXRos (mean intensity). (**C**) Representative confocal images of mito-QC VSMCs (white arrows show hyperfused mitochondria). Quantification of (**D**) mitochondrial length (µM) and (**E**) mitophagy index. Data shown as mean +/− S.E.M. (n = 3–6) *p < 0.05; ** *p*< 0.01; *** *p* < 0.001 compared to control. Scale bars = 10 μm.

Next, we examined whether p53 is essential for mitochondrial elongation and dysfunction during arterial calcification. Mitochondria within p53 knockdown VSMCs cultured in calcification medium showed reduced mitochondrial ROS (*p* < 0.05; Figure 6A) and enhanced mitochondrial membrane potential (*p* < 0.01; Figure 6B). Furthermore, mitochondria within p53 knockdown mito-QC VSMCs showed reduced mitochondrial length (3.4-fold decrease, *p* < 0.001; Figure 6C,D) with a concomitant increase in mitophagy index (3.1-fold increase, *p* < 0.05; Figure 6C,E) indicative of restored mitophagy, compared to mitochondria within scramble control cells. Altered mitochondrial length was also confirmed with TOM20 staining (Figure S2).

**Figure 7:**
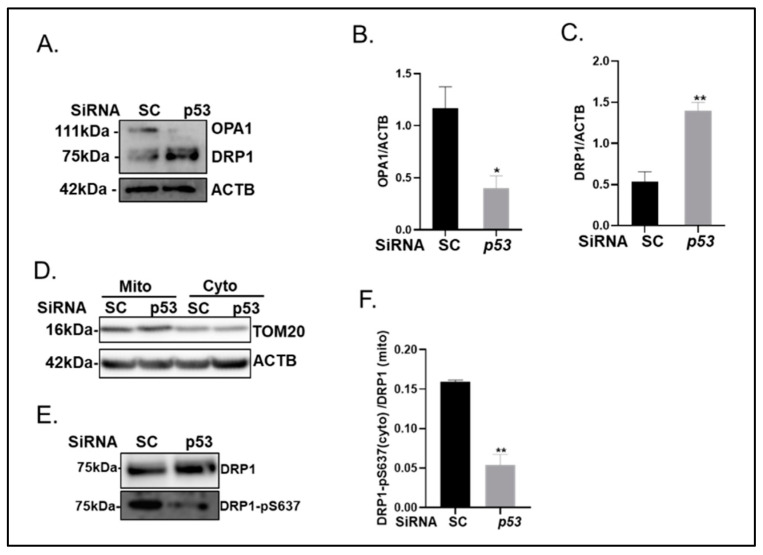
Knockdown of p53 in calcified VSMCs reverses Optic Atrophy Protein 1 (OPA1) and Dynamin-related protein 1 (DRP1) protein expression. VSMCs were transfected with either scrambled (SC) or p53 siRNA and cultured with high phosphate (3mM Pi) for up to 14 days. (**A**) Representative immunoblotting of whole cell lysates and associated quantification of (**B**) OPA1 and (**C**) DRP1 expression compared with ACTB (β-actin). (**D**) Representative immunoblotting of the mitochondrial and cytoplasmic fractions with TOM20 and ACTB from scrambled (SC) and p53 SiRNA transfected VSMCs. (**E**) Representative immunoblotting of mitochondrial expression of DRP1 and cytoplasmic expression of DRP1 -pS637 and associated quantification of (**F**) ratio of cytoplasmic DRP1- pS637/mitochondrial DRP1. Data shown as mean +/− S.E.M. (n = 3–6) * *p* < 0.05; ** *p* < 0.01 compared to control.

Consistent with these changes, immunoblotting studies revealed reduced expression of OPA1 (4.1-fold decrease, *p* < 0.05; Figure 7A,B) and increased expression of DRP1 (2.6-fold increase, *p* < 0.01; Figure 7A,C) in whole cell lysates produced from p53 knockdown VSMCs cultured in calcification medium versus scrambled control cells. Increased expression of DRP1 was also observed in the enriched mitochondrial fraction isolated from calcified VSMCs, conversely, the phosphorylation of cytoplasmic DRP1 Ser637 was significantly reduced (3.20-fold decrease, *p* < 0.001; Figure 7E,F) following p53 knockdown (Figure 7E) (enrichment confirmed by TOM20 expression, Figure 7D).

**Figure 8:**
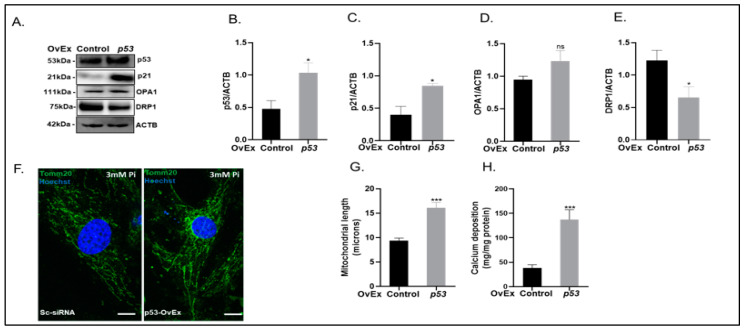
Overexpression of p53 enhances mitochondrial length, senescence and vascular calcification. VSMCs were transfected with either control or p53 vector (p53-OvEx) and cultured with high phosphate (3mM Pi) for up to 7 days. (**A**) Representative immunoblotting of whole cell lysates and associated quantification of (**B**) p53 and (**C**) p21 (**D**) Optic Atrophy Protein 1 (OPA1) expression compared with ACTB (β-actin) and (**E**) Dynamin-related protein 1 (DRP1). (**F**) Representative images of TOM20 staining (**G**) associated quantification of mitochondrial length. (**H**) Calcium content (mg/mg protein) of VSMCs. Data shown as mean +/− S.E.M. (n = 3–6) * *p*< 0.05; *** *p* < 0.001 compared to control.

In order to confirm our observation that p53 is essential for mitochondrial elongation and dysfunction during arterial calcification, we overexpressed p53 in VSMCs and subsequently calcified the cells for 7 days (Figure 8). Overexpression of p53 protein was confirmed by immunoblotting (2.2-fold increase, *p* < 0.05; Figure 8A,B). Overexpression of p53 induced an increase in the protein expression of p21 (2.6-fold increase, *p* < 0.05; Figure 8A,C) and a decrease in DRP1expression (2.1-fold decrease, *p* < 0.05; Figure 8A,E), with no further significant change in OPA1 expression (Figure 8A,D). A concomitant increase in mitochondrial length (2.1-fold increase, *p* < 0.001; Figure 8F,G) and VSMC calcification (3.7-fold increase, *p* < 0.001; Figure 8H) was also observed.

In conclusion, our data suggest that suppression of p53, a critical regulator of senescence, exerts protective effects against pathological calcification and restores mitochondrial dynamics by restoring DRP1-mediated fission required for mitophagic signalling in VSMCs.

## 3. Discussion

This study highlights the crucial role of mitochondrial function and cellular senescence in arterial calcification. Here, we provide novel functional evidence to suggest that p53 regulates this pathological process.

Cellular senescence is a critical mechanism that contributes to arterial calcification, a critical feature of vascular pathology in the aging population. Indeed, senescent VSMCs have been shown to calcify more readily than young, early passage VSMCs [29]. Typified as the cell cycle arrest of cells undergoing mitosis, cells activate p53 and p21 as final effectors for the senescence process [30]. Our data reveal that under calcifying culture conditions, murine VSMCs upregulate the expression of senescence markers, including p53, p21 and SA-β-gal, supporting and extending previous work in human cells and demonstrating that cellular senescence impacts vascular aging and arterial calcification [31]. Human VSMCs in culture can form multi-cellular nodules that spontaneously calcify and are positive for senescence-associated β-galactosidase [32]. Histological analysis of calcified arteries isolated from children on dialysis has revealed that VSMCs accumulate the senescence marker p21 [12]. Moreover, VSMCs cultured in vitro from these arteries underwent senescence at earlier passages than VSMCs from age-matched controls. Furthermore, VSMCs cultured from human calcified atherosclerotic plaques show a senescent phenotype [33], and it has been proposed that the senescence VSMCs could be the potential cause of plaque rupture [34].

Recent reports have shown that alterations in mitochondrial shape, size and function are important characteristics of cellular senescence and accelerate the senescent phenotype [25,35]. Furthermore, published data from our laboratory have demonstrated that calcified VSMCs show impaired respiratory status with reduced potential for ATP synthesis and reduced oxygen consumption rate [17]. Here, we highlight that under calcifying conditions, VSMCs have elongated mitochondria that avoid mitochondrial fission, which is required for their removal via mitophagy machinery. We propose that the elongated mitochondria evade mitophagy and sustain impaired mitochondria for a longer duration under calcifying conditions. This is further supported by our observation of reduced mitochondrial lysosomal delivery in calcified mito-QC VSMCs. Elongation may dilute the impaired respiratory status by equilibrating matrix metabolites and mitochondrial membrane components [36], thus exerting a protective action against cell death via apoptosis [37].

To understand the mechanisms underpinning mitochondrial elongation, we determined the expression levels of fusion and fission proteins, as increased mitochondrial fission is essential for apoptosis and mitophagy [37], whereas elongation (fusion) of mitochondria has been noted in cellular senescence models [25,38,39]. We show that mitochondrial elongation in calcified VSMCs is driven not only by the loss of DRP1, a key regulator of mitochondrial fission required for mitophagic degradation [40], but also enhanced mitochondrial fusion via the inhibitory phosphorylation of DRP1. Interestingly, our results are in contrast to previous data suggesting that DRP1 inhibition can diminish VSMC mitochondrial damage and calcification [41]; however, this previous work did not explore the regulation of DRP1 function by post-translational modifications. Further studies are now required to directly test the functional role of key regulators of mitochondrial function in VSMC calcification. These include the mitochondrial fusion/fission proteins OPA1, DRP1 and MFN1/2 [42], as well as PINK-1 and Parkin, which activate the clearance of damaged mitochondria [43].

Previous studies highlighting a critical role for the tumour suppressor p53 in mitochondrial function are supported by our findings that p53 knockdown in VSMCs leads to reduced mitochondrial length, restored mitophagy and enhanced membrane potential. Previously, p53 has been shown to promote mitochondrial respiration via cytochrome c oxidase 2 transcriptional activation [44]. Furthermore, p53 directly cooperates with the Bcl-2 protein family within the mitochondrial outer membrane during a genotoxic response [45]. Additionally, it has been shown that p53 directly modulates mitochondrial function in a human endometrial carcinoma cell-line, whereby infection with the p53 adenovirus induced a significant decrease in mitochondrial membrane potential and induced mitochondrial fusion preceding cellular senescence [15]. Indeed, it has been demonstrated that p53 activation and over-expression promote cellular senescence in a range of cells, including human bladder carcinoma cells and fibroblasts [15,46]. These findings are further supported by our observation of increased expression of the senescence regulator p21 following p53 overexpression in calcifying VSMCs. In addition, p16 ^ink4a^ has been reported to play an important role in proliferation arrest and cellular senescence [47]. However, in the present study, no changes in p16 ^ink4a^ expression levels were observed during VSMC calcification. These data likely reflect previous findings demonstrating that senescence induced by p16 ^ink4a^ is mostly regulated by environmental factors such as ionizing radiation, DNA-damaging agents and oncogene-mediated activation [48,49].

In contrast to our data, recent studies have reported reduced expression of p53 during VSMC calcification following treatment of cells with the putative cancer therapeutics, piperlongumine [50] and teniposide [51]. These divergent observations may reflect key differences in the species, passage status and culture conditions of the cells investigated. Furthermore, these findings likely reflect the activation of different signalling mechanisms, with piperlongumine modulating PTEN transcription [50] and teniposide regulating the miR-203-3p-BMP2 pathway [51]. Indeed, our data support novel findings challenging the commonly accepted paradigm of p53 having primarily pro-apoptotic functions in VSMCs [52].

A number of studies have revealed an important role for p53 overexpression in the induction of senescence and alteration of mitochondrial dynamics in a variety of cell types. It has been shown that p53 directly modulates the mitochondrial function in a human endometrial carcinoma cell-line, whereby infection with p53 adenovirus induced a significant decrease in mitochondrial membrane potential and induced mitochondrial fusion preceding cellular senescence [15,53]. Furthermore, it has been demonstrated that p53 activation and overexpression promote cellular senescence in a range of cells, including human bladder carcinoma cells and fibroblasts [15,46]. Future functional studies in non-calcified VSMCs would therefore be highly informative in clarifying whether calcification is a cause or consequence of altered p53 levels.

Our data further show that p53 induces mitochondrial elongation in VSMC calcification through a novel post-translational mechanism, mediating DRP1 Ser637 phosphorylation. Indeed, we observed reduced localisation of DRP1 in mitochondria, likely reflecting the previous reports that the phosphorylation status of Ser637 plays a dominant role in the subcellular localisation of DRP1 [54]. A comparable p53/DRP1-mediated mechanism has also been reported to induce cellular senescence in cancer cell lines [15], highlighting the importance of this pathway in different pathologies.

In summary, we demonstrate that VSMC calcification promotes notable mitochondrial elongation and cellular senescence via Drp1 phosphorylation. Furthermore, this work indicates that p53-induced mitochondrial fusion underpins cellular senescence by reducing mitochondrial function.

## 4. Materials and Methods

### 4.1. Maintenance of Mito-QC Mice

The generation and characterisation of the mito-QC mouse was previously described [22]. As described below, 2–3-month-old mito-QC mice were used for VSMC cultures. Mice were euthanised by CO_2_ followed by cervical dislocation. The mito-QC mouse work was approved by University of Edinburgh ethics committee.

### 4.2. VSMC Isolation, Culture and Calcification

Primary aortic VSMCs were obtained and calcification induced as described previously using protocols established by our laboratory [18]. Cells were cultured for up to 14 days, and the medium changed every two or three days. For all the experiments, n = 3 is representative of 3 independent experiments pooled from 8eight aortas. Calcium deposition was assessed by HCl leaching [18].

### 4.3. Transfection Assays

Cells were transfected with 80 pmol murine p53 (SC-29436; Santa Cruz Biotechnology, Dallas, TX, USA) or scrambled control siRNA (SC-37007; Santa Cruz Biotechnology) with siRNA transfection reagent (SC-29528; Santa Cruz Biotechnology) following the manufacturer’s protocol. For p53 overexpression, Trp53_OMu22609D_pcDNA3.1+/C-(K)-DYK, Clone ID:OMu22609D was transfected in VSMCs at a concentration of 2 μg/mL. Vector without Trp53 ORF was used as a control.

### 4.4. Cell Imaging

Cells were seeded in 24-well plates (5 × 10^4^ cells per well) on glass cover slips and subsequently fixed with 3.7% paraformaldehyde (PFA) (pH 7.0) at 4 °C, permeabilised with 0.1% triton X100 (Sigma, St. Louis, MO, USA) and blocked with 2% goat before incubating with primary antibodies TOM20 (1:300; 42406; Cell Signaling Technology, Danvers, MA, USA) overnight. After washing, cells were incubated for 1 h in the dark with Alexa Fluor@488 anti-rabbit antibody (A11034; Life Technologies, Waltham, MA, USA). Hoechst staining was then performed (1:10,000; Sigma). Glass coverslips were mounted onto slides using Prolong^®^Gold Anti-Fade Reagent with DAPI (Life Technologies) and the fluorescence signal observed (Zeiss LSM 710; Oberkochen, Germany).

### 4.5. Senescence Associated β-Gal Staining

Senescence-associated β–galactosidase (SA β-gal) staining was undertaken according to the manufacturers’ instructions (Merk Millipore, Burlington, MA, USA). Briefly, cells were fixed (0.25% glutaraldehyde) and SA-β-gal staining (pH 6.0) was carried out. Images were captured using an Axiovert 25 (Zeiss) microscope. SA-β-gal positive cells were quantified using image analysis software (ImageJ, Bethesda, MD, USA).

### 4.6. MitoTracker Staining

Cells were stained with 100 nM MitoTracker Red CMXRos (Invitrogen, Waltham, MA, USA). Subsequently, cells were fixed in 4% PFA (pH7), images of the stained cells were captured (Zeiss LSM 710; Oberkochen, Germany) and staining quantified using Image J.

### 4.7. Measurement of ROS Levels

ROS production in cultured cells was determined using a commercially available mitochondrial ROS detection kit (Cayman Chemicals, Ann Arbor, MI, USA) with an excitation wavelength of 480–515 nm and emission wavelength at 560–600 nm.

### 4.8. Mitophagy Assay

Mito-QC VSMCs were fixed at 4 °C with 4% PFA (pH7). Images of the stained cells were captured (Zeiss LSM 710; Oberkochen, Germany). Red/mCherry puncta, indicative of mitophagy index, were counted (mitoQC counter, Fiji plugin) and organelle length measured using image analysis software (ImageJ, WI, USA).

### 4.9. Mitochondrial Length Measurement

Mitochondrial length measurement was quantified with image analysis software (ImageJ, WI, USA).

### 4.10. Analysis of Gene Expression

RNA was extracted and reverse transcribed, and specific cDNAs were quantified by real-time qPCR [5]. The following mouse primers (Sigma) were used: p53 Forward 5′-GTATTTCACCCTCAAGATCC, Reverse 5′-TGGGCATCCTTTAACTCTA; p21 Forward 5′-CCTGGTGATGTCCGACCTG, Reverse 5′-CCATGAGCGCATCGAATC.

### 4.11. Immunoblotting

Cells were lysed in radioimmunoprecipitation assay (RIPA) buffer containing Protease Inhibitor Cocktail (Thermo Fisher Scientific, Waltham, MA, USA) and total protein concentration measured using a commercially available BCA protein assay (Thermo Scientific). Mitochondrial fractions were isolated from cultured VSMCs using a commercial kit (89874; Thermo Scientific). Immunoblotting was performed as previously described [55]. Nitrocellulose membranes were incubated for up to 16 h at 4 °C with primary antibodies (1:1000 dilution in 5% milk in TBST), p53 (7F5; Cell Signaling Technology), p21 (2947; Cell Signaling Technology), TOM20 (DBT4N; Cell Signaling Technology), OPA1 (ab157457; Abcam, Cambridge, UK), DRP1 (ab184247; Abcam), DRP1-pS637 (BT-PHS00841; Bioassay Technology Laboratory, Birmingham, UK), TNAP (MAB 2909; R & D Systems, Minneapolis, MN, USA) and OCN (ab93876; Abcam). Membranes were then probed with HRP-conjugated goat anti-rabbit IgG (P0448; Dako) and goat anti-rat IgG (HAF005; Dako, Glostrup, Denmark) for 1 h. Membranes were developed using the GeneGenome system (Syngene, Frederick, MD, USA). Membranes were then washed and re-probed for mouse β-actin expression (1:10,000; A3845; Sigma). Semi-quantitative assessment of band intensity was performed using ImageJ image analysis software.

### 4.12. Statistical Analysis

All data are presented as mean ± SEM. Data were analysed by unpaired *t*-test or one-way analysis of variance (ANOVA) followed by Tukey’s range test. All statistical analysis was performed using GraphPad prism (CA, USA). Values of *p* < 0.05 were regarded as significant, and *p* values are shown as: * *p* < 0.05; ** *p* < 0.01; *** *p* < 0.001.

## Data Availability

The data presented in this study are available within the article or Supplementary Materials. For the purpose of open access, the authors have applied a Creative Commons Attribution (CC BY) licence to any author accepted manuscript version arising from this submission.

## Supplementary Materials

The following supporting information can be downloaded at: https://www.mdpi.com/article/10.3390/ijms24021643/s1.
