# Supplementary material for: p53 Regulates Mitochondrial Dynamics in Vascular Smooth Muscle Cell Calcification
Source: Int J Mol Sci. 2023 Jan 13;24(2):1643. doi: 10.3390/ijms24021643 (PMC9864220; doi:10.3390/ijms24021643)
Supplement: Supplementary file 1 [file ijms-24-01643-s001.zip › ijms-1984129-supplementary.pptx]

## Slide 1
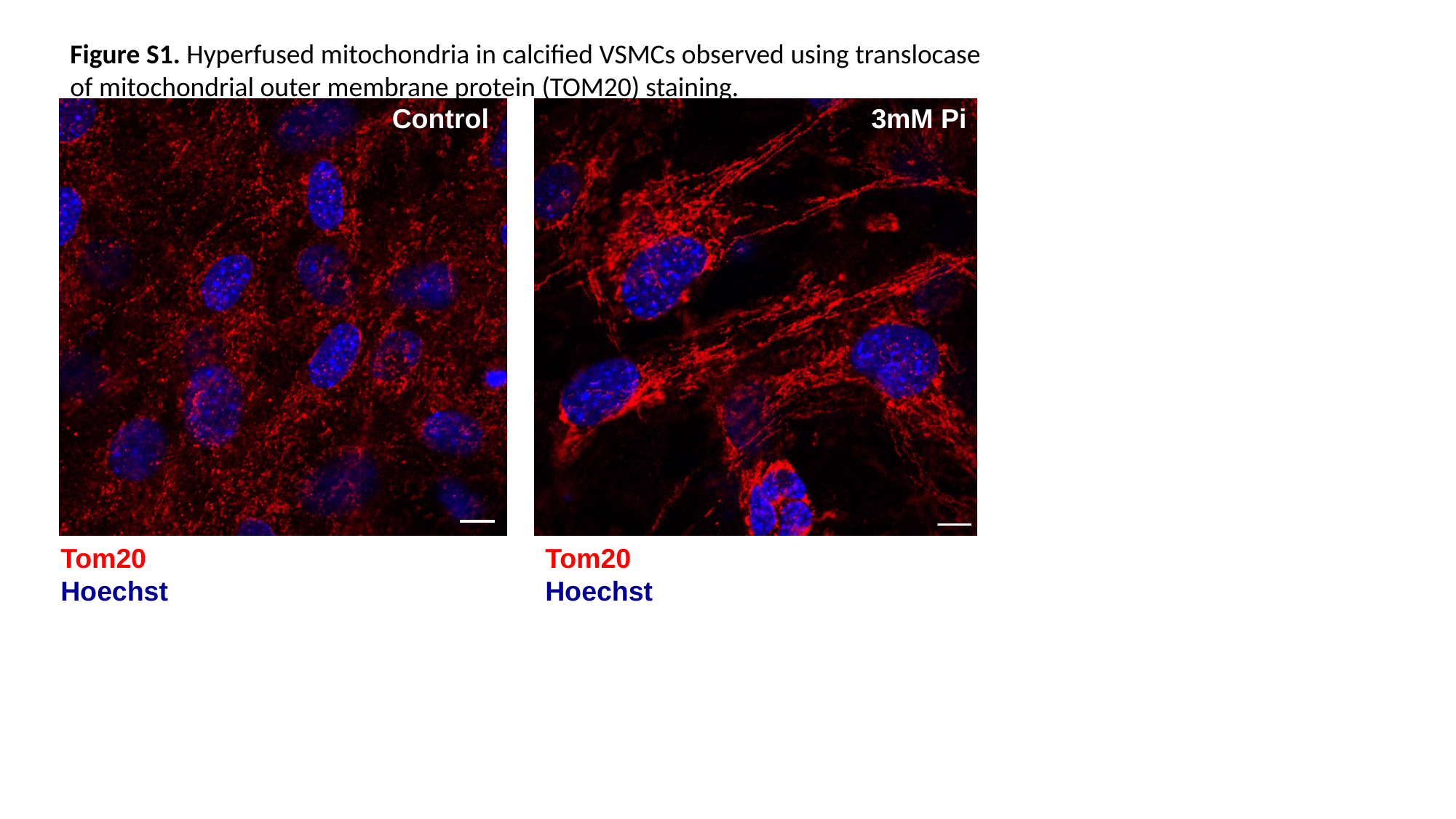

Figure S1. Hyperfused mitochondria in calcified VSMCs observed using translocase of mitochondrial outer membrane protein (TOM20) staining.
Control
3mM Pi
Tom20
Hoechst
Tom20
Hoechst

## Slide 2
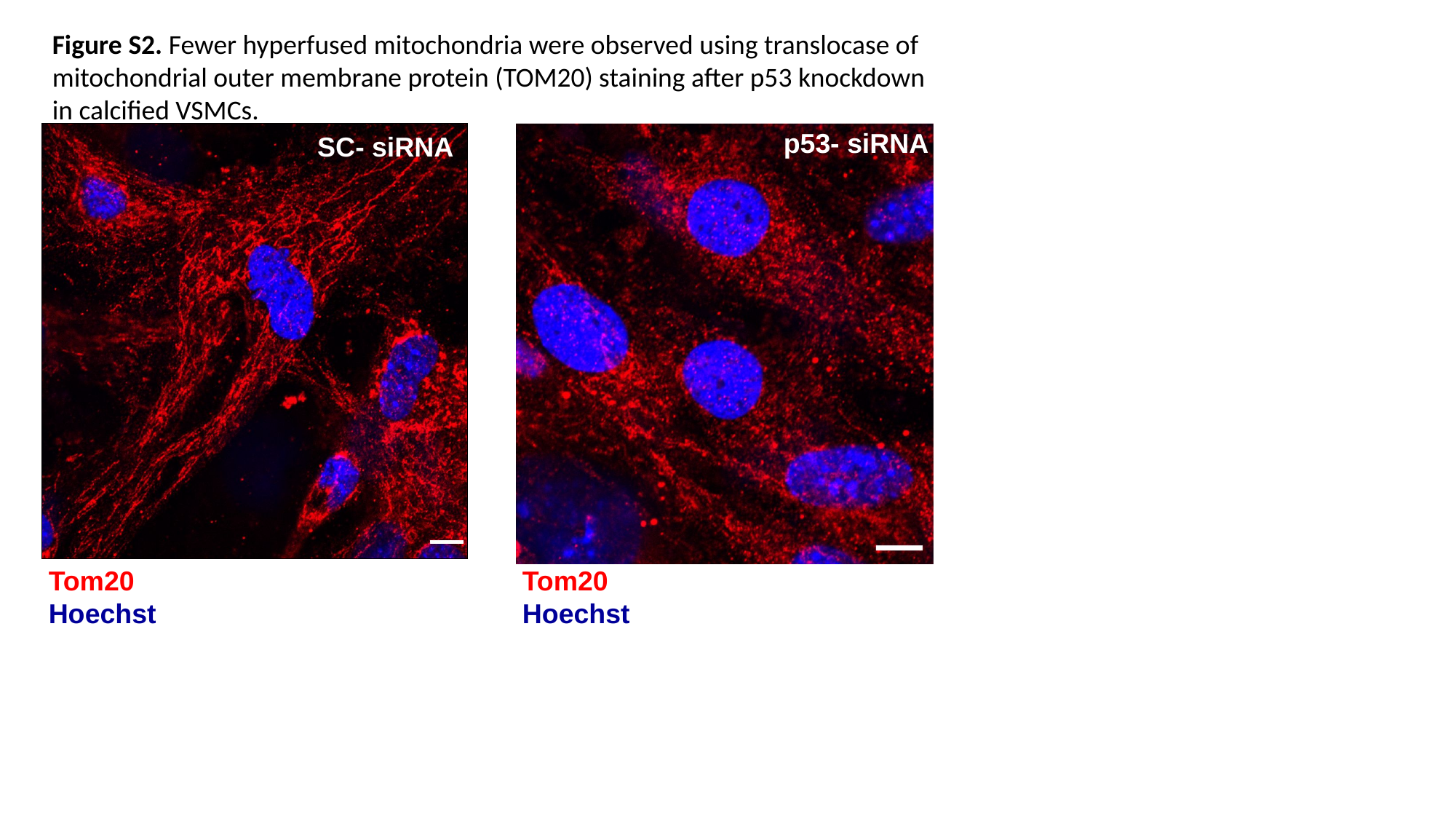

Figure S2. Fewer hyperfused mitochondria were observed using translocase of mitochondrial outer membrane protein (TOM20) staining after p53 knockdown in calcified VSMCs.
 p53- siRNA
 SC- siRNA
Tom20
Hoechst
Tom20
Hoechst

## Slide 3
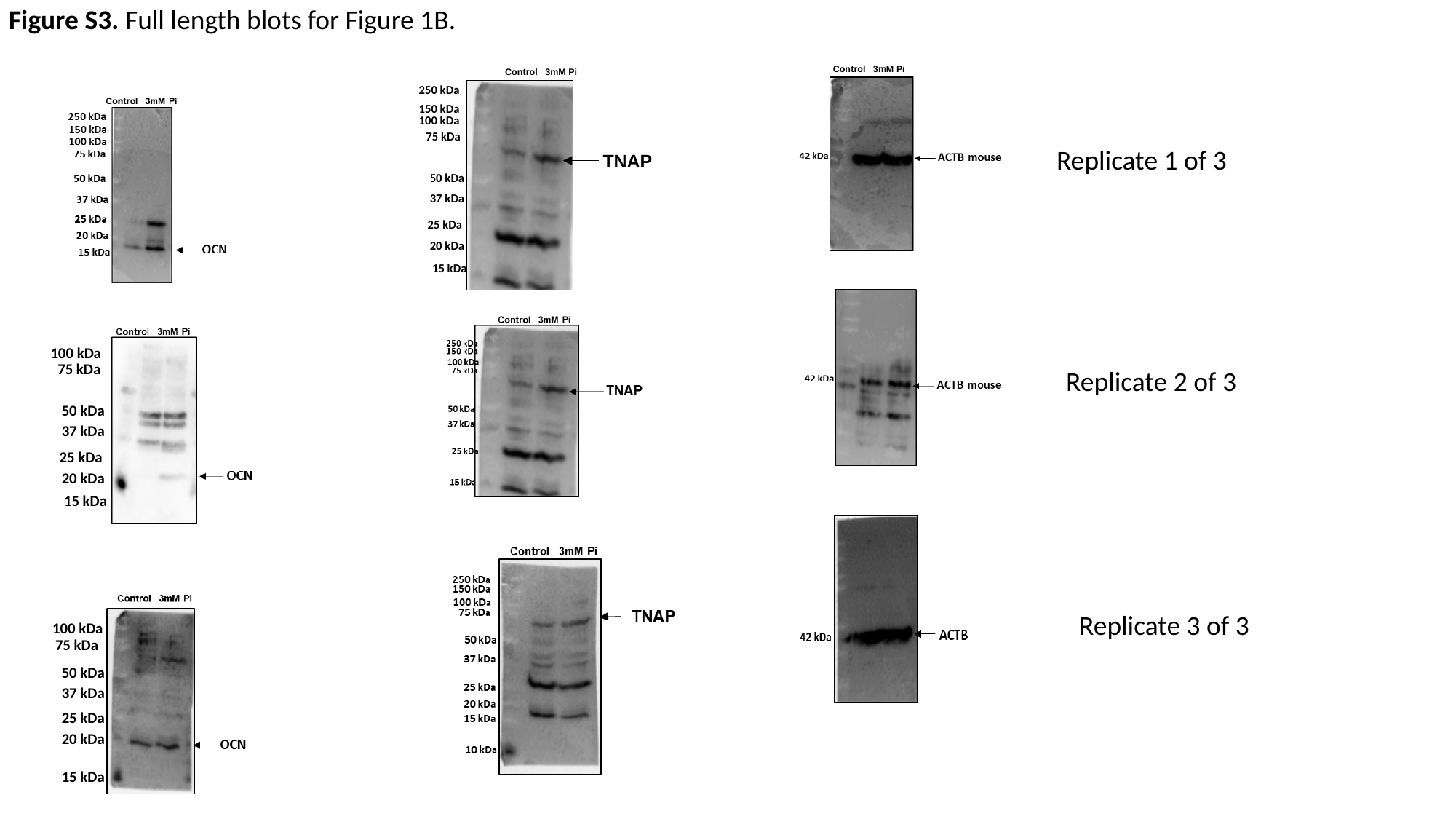

Figure S3. Full length blots for Figure 1B.
Control 3mM Pi
Control 3mM Pi
TNAP
250 kDa
150 kDa
100 kDa
75 kDa
50 kDa
37 kDa
25 kDa
20 kDa
15 kDa
Replicate 1 of 3
100 kDa
75 kDa
50 kDa
37 kDa
25 kDa
20 kDa
15 kDa
Replicate 2 of 3
100 kDa
75 kDa
50 kDa
37 kDa
25 kDa
20 kDa
15 kDa
Replicate 3 of 3

## Slide 4
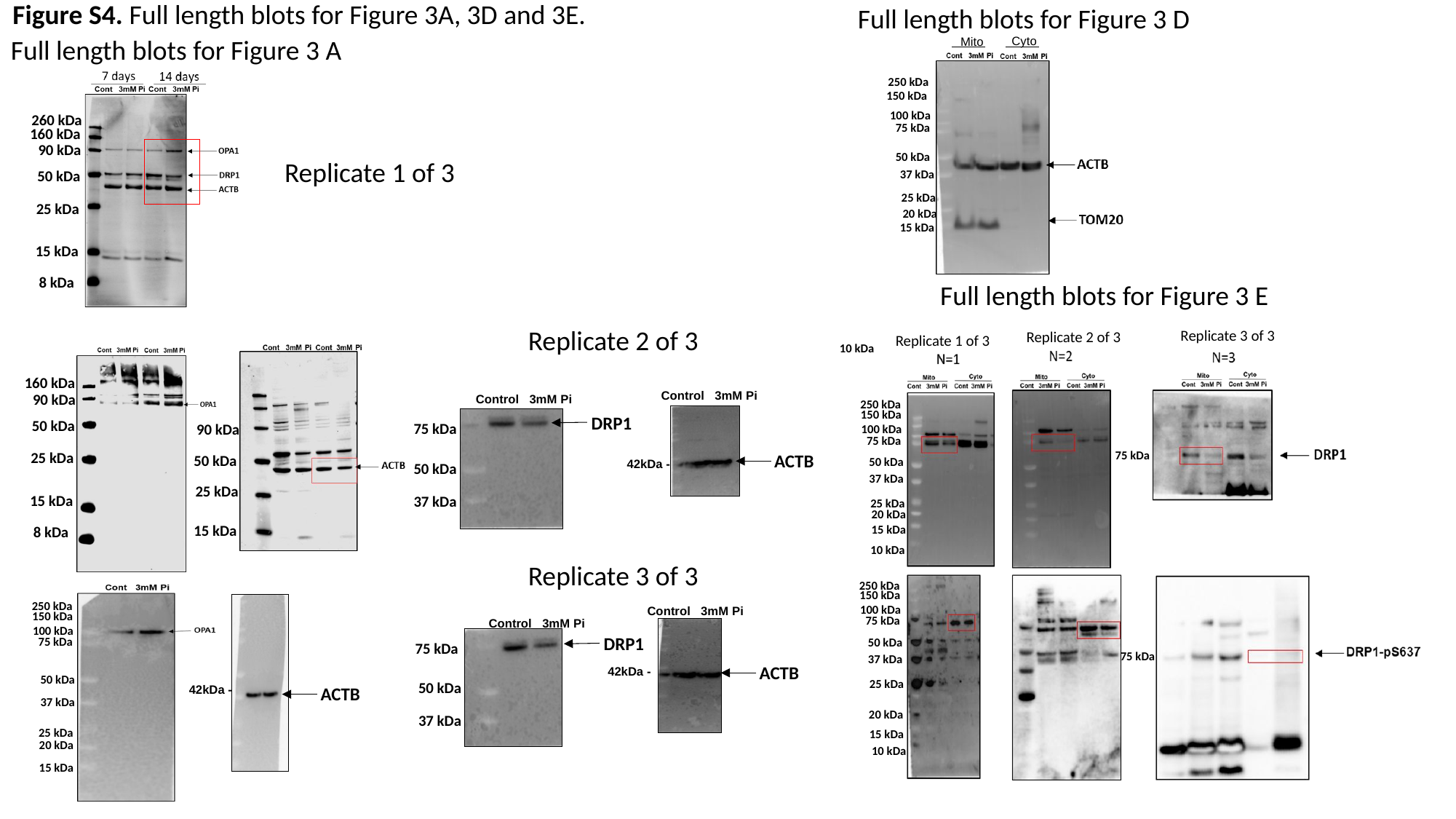

Figure S4. Full length blots for Figure 3A, 3D and 3E.
Full length blots for Figure 3 D
Full length blots for Figure 3 A
Cyto
Mito
250 kDa
150 kDa
 100 kDa
75 kDa
50 kDa
37 kDa
25 kDa
15 kDa
10 kDa
20 kDa
260 kDa
90 kDa
50 kDa
25 kDa
15 kDa
 8 kDa
160 kDa
Replicate 1 of 3
Full length blots for Figure 3 E
Replicate 2 of 3
Replicate 3 of 3
Replicate 2 of 3
Replicate 1 of 3
50 kDa
160 kDa
Control 3mM Pi
90 kDa
Control 3mM Pi
250 kDa
 150 kDa
 100 kDa
75 kDa
50 kDa
37 kDa
25 kDa
15 kDa
20 kDa
DRP1
75 kDa
50 kDa
37 kDa
ACTB
42kDa -
50 kDa
90 kDa
75 kDa
25 kDa
25 kDa
15 kDa
15 kDa
 8 kDa
10 kDa
Replicate 3 of 3
250 kDa
 150 kDa
 100 kDa
75 kDa
50 kDa
37 kDa
25 kDa
15 kDa
10 kDa
250 kDa
 150 kDa
 100 kDa
75 kDa
50 kDa
37 kDa
25 kDa
15 kDa
ACTB
Control 3mM Pi
Control 3mM Pi
DRP1
ACTB
42kDa -
75 kDa
75 kDa
50 kDa
42kDa -
20 kDa
37 kDa
20 kDa

## Slide 5
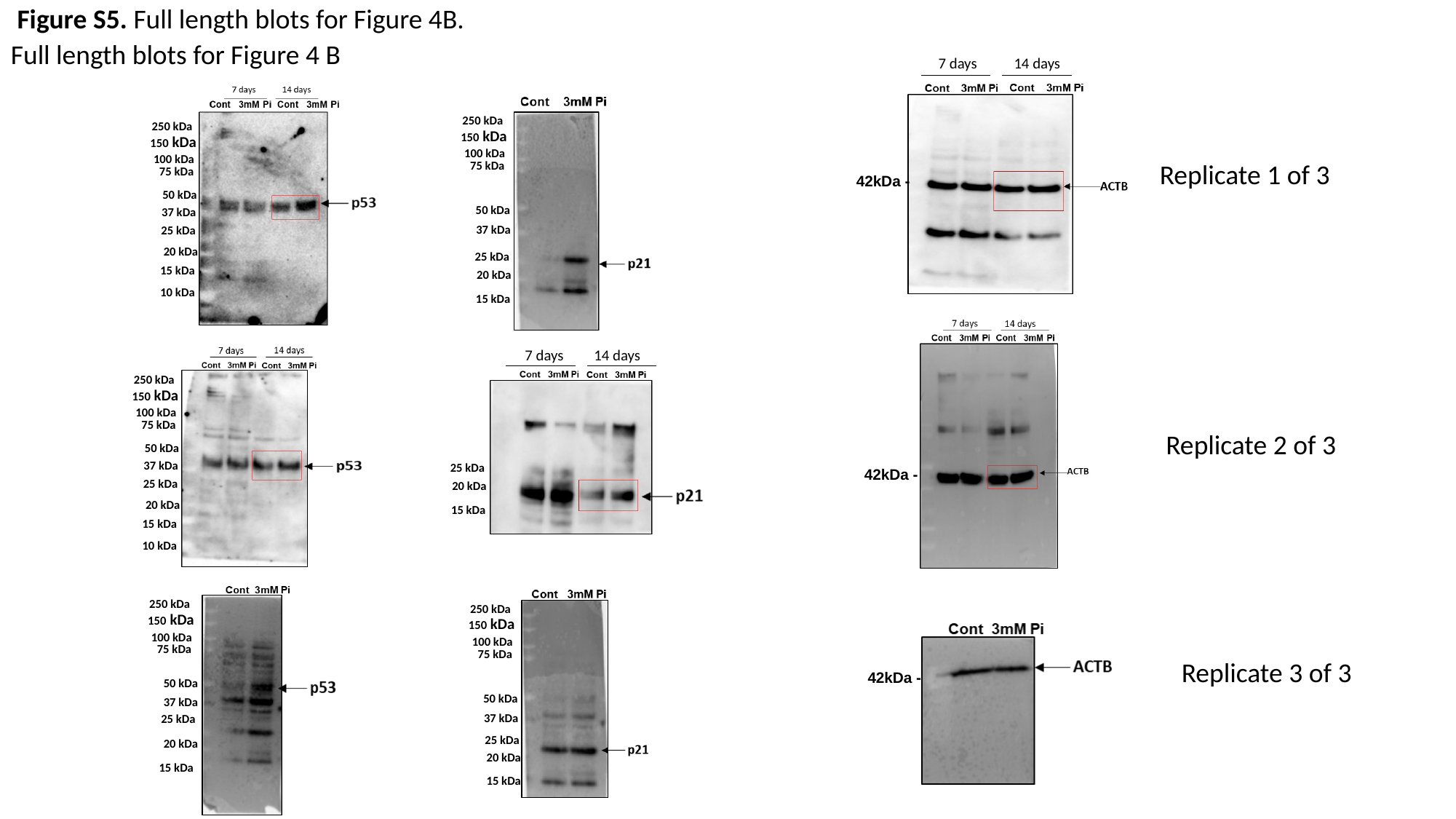

Figure S5. Full length blots for Figure 4B.
Full length blots for Figure 4 B
7 days
14 days
42kDa -
250 kDa
150 kDa
 100 kDa
75 kDa
50 kDa
37 kDa
25 kDa
15 kDa
20 kDa
250 kDa
150 kDa
 100 kDa
75 kDa
50 kDa
37 kDa
25 kDa
15 kDa
10 kDa
20 kDa
Replicate 1 of 3
42kDa -
7 days
14 days
250 kDa
150 kDa
 100 kDa
75 kDa
50 kDa
37 kDa
25 kDa
15 kDa
10 kDa
20 kDa
Replicate 2 of 3
25 kDa
20 kDa
15 kDa
250 kDa
150 kDa
 100 kDa
75 kDa
50 kDa
37 kDa
25 kDa
15 kDa
20 kDa
250 kDa
150 kDa
 100 kDa
75 kDa
50 kDa
37 kDa
25 kDa
15 kDa
20 kDa
42kDa -
Replicate 3 of 3

## Slide 6
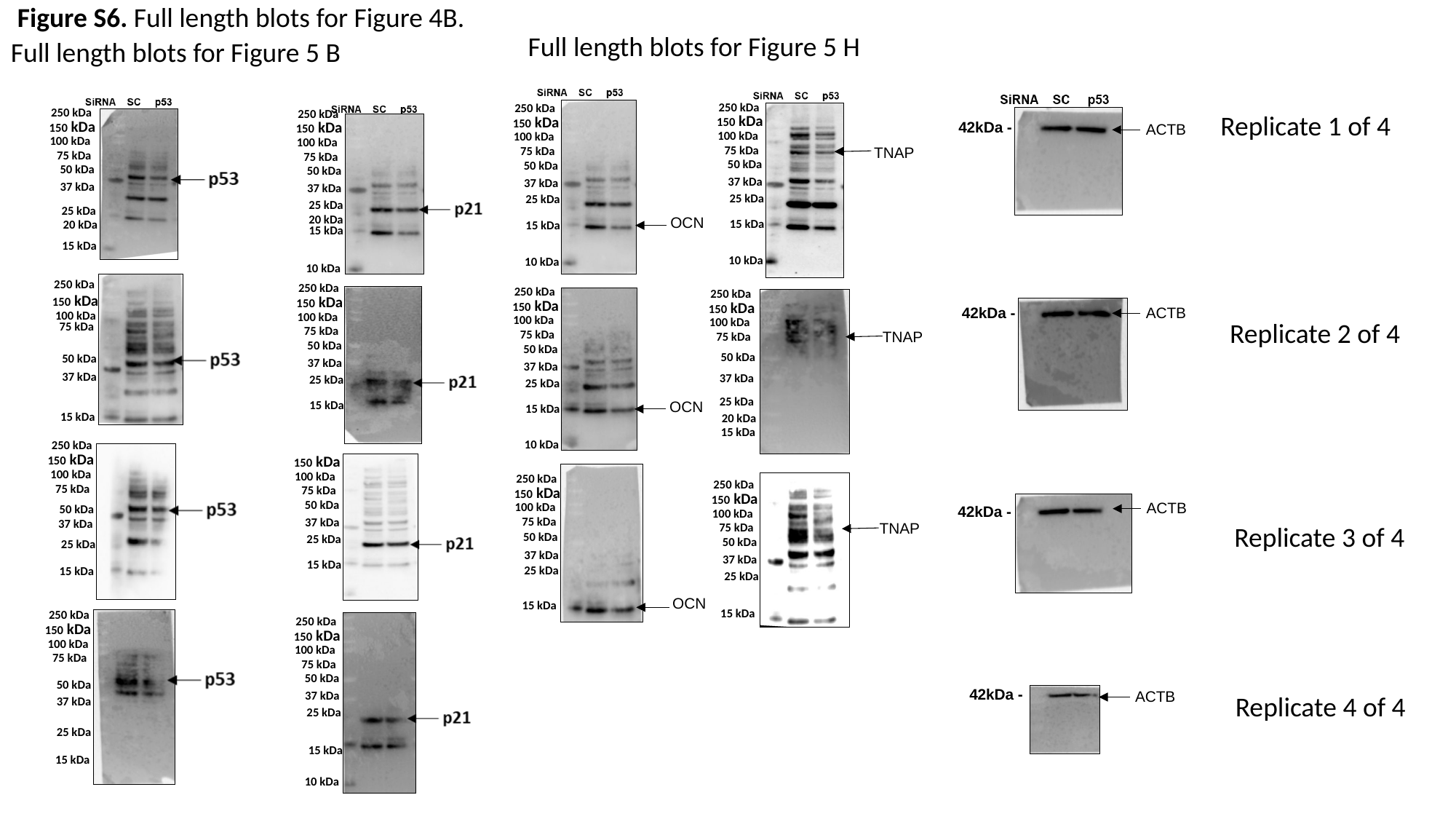

Figure S6. Full length blots for Figure 4B.
Full length blots for Figure 5 H
Full length blots for Figure 5 B
OCN
TNAP
TNAP
TNAP
42kDa -
ACTB
250 kDa
150 kDa
 100 kDa
75 kDa
50 kDa
37 kDa
25 kDa
15 kDa
10 kDa
250 kDa
150 kDa
 100 kDa
75 kDa
50 kDa
37 kDa
25 kDa
15 kDa
10 kDa
250 kDa
150 kDa
 100 kDa
75 kDa
50 kDa
37 kDa
25 kDa
15 kDa
250 kDa
150 kDa
 100 kDa
75 kDa
50 kDa
37 kDa
25 kDa
15 kDa
10 kDa
Replicate 1 of 4
20 kDa
20 kDa
250 kDa
150 kDa
 100 kDa
75 kDa
50 kDa
37 kDa
250 kDa
150 kDa
 100 kDa
75 kDa
50 kDa
37 kDa
25 kDa
15 kDa
250 kDa
150 kDa
 100 kDa
75 kDa
50 kDa
37 kDa
25 kDa
15 kDa
10 kDa
250 kDa
150 kDa
 100 kDa
75 kDa
50 kDa
37 kDa
25 kDa
20 kDa
15 kDa
OCN
ACTB
42kDa -
Replicate 2 of 4
15 kDa
250 kDa
150 kDa
 100 kDa
75 kDa
50 kDa
37 kDa
25 kDa
15 kDa
150 kDa
 100 kDa
75 kDa
50 kDa
37 kDa
25 kDa
15 kDa
OCN
250 kDa
150 kDa
 100 kDa
75 kDa
50 kDa
37 kDa
25 kDa
15 kDa
250 kDa
150 kDa
 100 kDa
75 kDa
50 kDa
37 kDa
25 kDa
15 kDa
ACTB
42kDa -
Replicate 3 of 4
250 kDa
150 kDa
 100 kDa
75 kDa
50 kDa
37 kDa
25 kDa
15 kDa
250 kDa
150 kDa
 100 kDa
75 kDa
50 kDa
37 kDa
25 kDa
15 kDa
10 kDa
42kDa -
ACTB
Replicate 4 of 4

## Slide 7
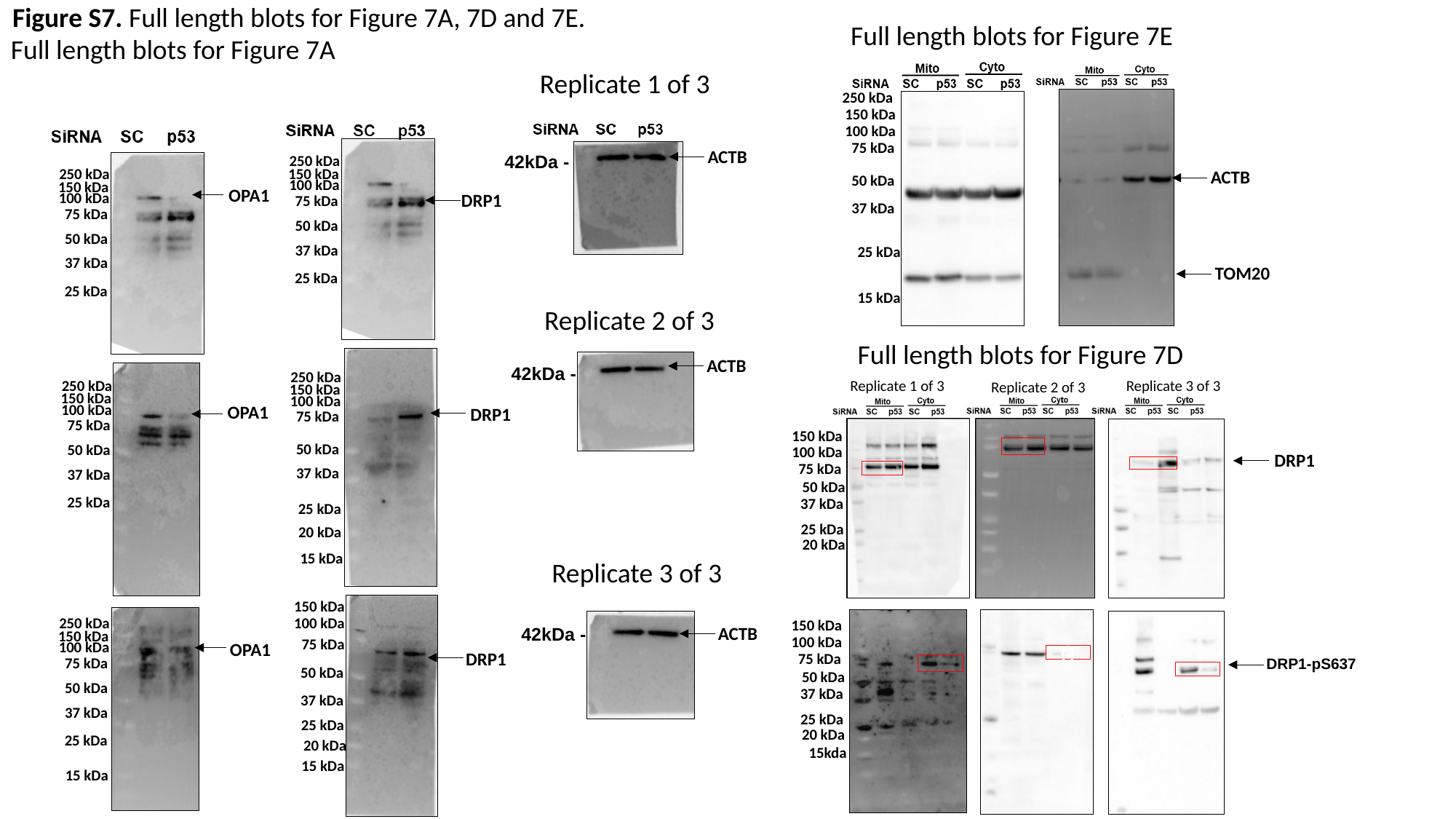

Figure S7. Full length blots for Figure 7A, 7D and 7E.
Full length blots for Figure 7E
Full length blots for Figure 7A
Replicate 1 of 3
250 kDa
150 kDa
100 kDa
75 kDa
50 kDa
37 kDa
25 kDa
15 kDa
ACTB
42kDa -
DRP1
250 kDa
150 kDa
100 kDa
75 kDa
50 kDa
37 kDa
25 kDa
OPA1
250 kDa
150 kDa
100 kDa
75 kDa
50 kDa
37 kDa
25 kDa
ACTB
TOM20
Replicate 2 of 3
Full length blots for Figure 7D
DRP1
ACTB
42kDa -
250 kDa
150 kDa
100 kDa
75 kDa
50 kDa
37 kDa
25 kDa
OPA1
250 kDa
150 kDa
100 kDa
75 kDa
50 kDa
37 kDa
25 kDa
Replicate 1 of 3
Replicate 3 of 3
Replicate 2 of 3
150 kDa
100 kDa
75 kDa
50 kDa
37 kDa
25 kDa
20 kDa
DRP1
20 kDa
15 kDa
Replicate 3 of 3
150 kDa
100 kDa
75 kDa
50 kDa
37 kDa
25 kDa
20 kDa
15 kDa
DRP1
OPA1
250 kDa
150 kDa
100 kDa
75 kDa
50 kDa
37 kDa
25 kDa
0
150 kDa
100 kDa
75 kDa
50 kDa
37 kDa
25 kDa
20 kDa
 15kda
ACTB
42kDa -
DRP1-pS637
15 kDa

## Slide 8
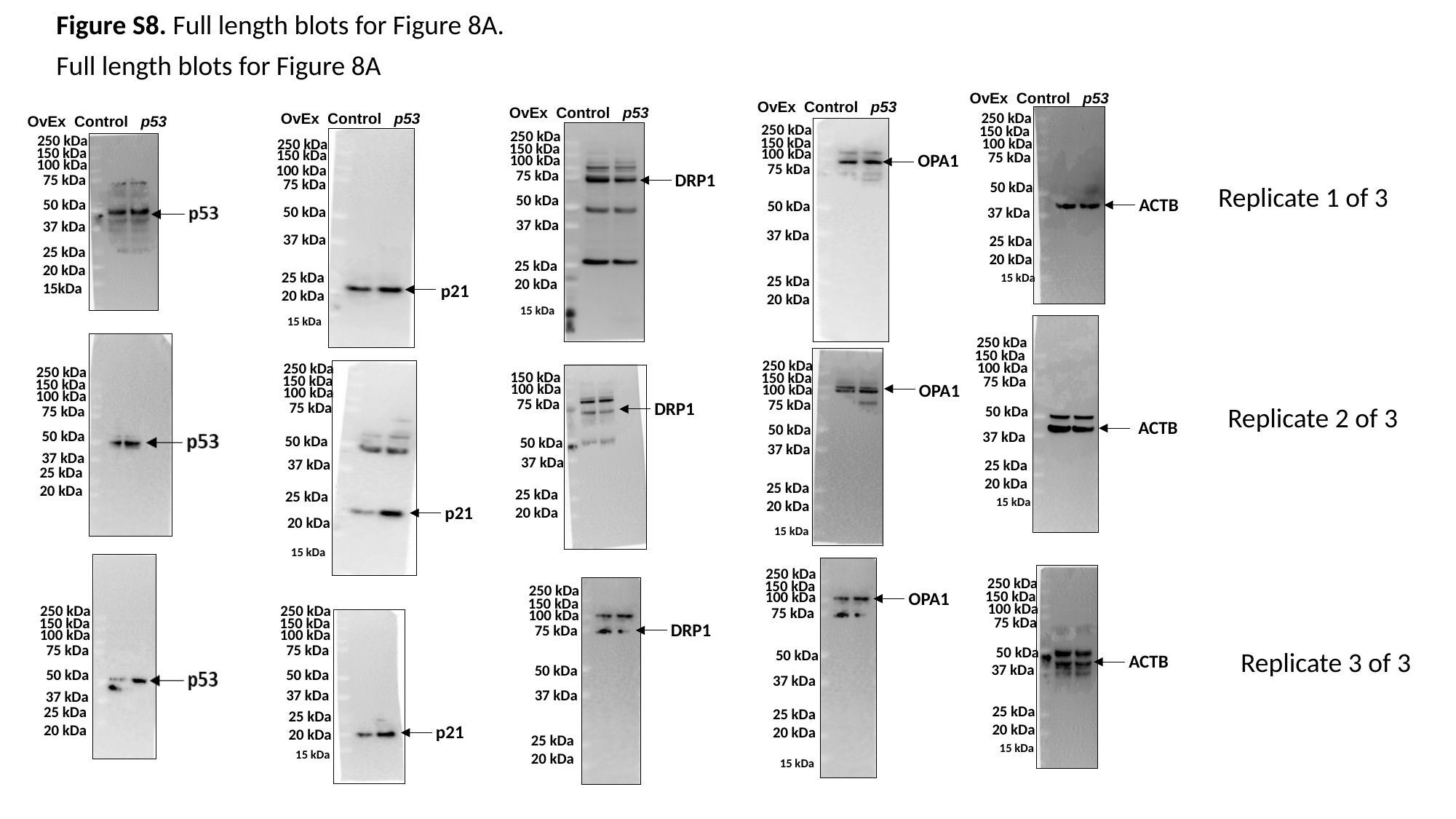

Figure S8. Full length blots for Figure 8A.
Full length blots for Figure 8A
 OvEx Control p53
 OvEx Control p53
 OvEx Control p53
250 kDa
150 kDa
100 kDa
75 kDa
50 kDa
37 kDa
25 kDa
20 kDa
15 kDa
 OvEx Control p53
ACTB
ACTB
ACTB
 OvEx Control p53
250 kDa
150 kDa
100 kDa
75 kDa
50 kDa
37 kDa
25 kDa
20 kDa
OPA1
OPA1
OPA1
250 kDa
150 kDa
100 kDa
75 kDa
50 kDa
37 kDa
25 kDa
20 kDa
15 kDa
DRP1
250 kDa
150 kDa
100 kDa
75 kDa
50 kDa
37 kDa
25 kDa
20 kDa
15kDa
p21
250 kDa
150 kDa
100 kDa
75 kDa
50 kDa
37 kDa
25 kDa
20 kDa
15 kDa
Replicate 1 of 3
250 kDa
150 kDa
100 kDa
75 kDa
50 kDa
37 kDa
25 kDa
20 kDa
15 kDa
250 kDa
150 kDa
100 kDa
75 kDa
50 kDa
37 kDa
25 kDa
20 kDa
15 kDa
250 kDa
150 kDa
100 kDa
75 kDa
50 kDa
37 kDa
25 kDa
15 kDa
250 kDa
150 kDa
100 kDa
75 kDa
50 kDa
37 kDa
25 kDa
20 kDa
p21
150 kDa
100 kDa
75 kDa
50 kDa
37 kDa
25 kDa
20 kDa
DRP1
Replicate 2 of 3
20 kDa
250 kDa
150 kDa
100 kDa
75 kDa
50 kDa
37 kDa
25 kDa
20 kDa
15 kDa
250 kDa
150 kDa
100 kDa
75 kDa
50 kDa
37 kDa
25 kDa
20 kDa
15 kDa
250 kDa
150 kDa
100 kDa
75 kDa
50 kDa
37 kDa
25 kDa
20 kDa
DRP1
250 kDa
150 kDa
100 kDa
75 kDa
50 kDa
37 kDa
25 kDa
20 kDa
250 kDa
150 kDa
100 kDa
75 kDa
50 kDa
37 kDa
25 kDa
20 kDa
15 kDa
p21
Replicate 3 of 3
